# The Effect of Splenectomy on Postoperative Morbidity and Survival in Patients with Peritoneal Carcinomatosis

**DOI:** 10.3390/jcm14228223

**Published:** 2025-11-20

**Authors:** Serkan Ademoğlu, İsa Caner Aydın, Ahmet Orhan Sunar, Mehmet Ömer Özduman, Uğur Duman, Mürşit Dinçer, Erdal Polat, Mustafa Duman

**Affiliations:** 1 Gastroenterological Surgery Department, Kosuyolu Yüksek İhtisas Training and Research Hospital, TR University of Health Sciences, Istanbul 34865, Türkiye; 2 Gastroenterological Surgery Department, Zonguldak Ataturk State Hospital, Ministry of Health, Zonguldak 67100, Türkiye; 3 General Surgery Department, Bursa City Hospital, TR University of Health Sciences, Bursa 16250, Türkiye

**Keywords:** cytoreductive surgery, hyperthermic intraperitoneal chemotherapy, splenectomy, peritoneal carcinomatosis

## Abstract

**Background/Objectives**: Cytoreductive surgery (CRS) and hyperthermic intraperitoneal chemotherapy (HIPEC) are effective treatment modalities for patients with peritoneal carcinomatosis (PC), though splenectomy is frequently required and its impact on outcomes remains unclear. Previous studies have evaluated splenectomy as a binary variable without distinguishing surgical indication, potentially obscuring important prognostic differences. This retrospective study aimed to evaluate the impact of splenectomy on postoperative morbidity and survival in patients undergoing CRS + HIPEC. **Methods**: A retrospective analysis was conducted on 149 patients who underwent CRS + HIPEC between 2018–2022 at a single tertiary center. The study examined patients with various cancer origins, including colorectal, ovarian, gastric, pseudomyxoma peritonei, and malignant peritoneal mesothelioma. Demographic characteristics, surgical procedures, complications, and survival outcomes were comprehensively analyzed. Patients were categorized by splenectomy and further stratified by surgical indication (iatrogenic injury, peritoneal implants, hilar tumor invasion). **Results**: Splenectomy was associated with longer ICU stay (median 1.5 vs. 1 day, *p* < 0.001) and hospitalization (median 12 vs. 9 days, *p* = 0.005). Individual pulmonary complications were more frequent in the splenectomy group, though major complication rates (Clavien–Dindo ≥ 3) were similar (34.7% vs. 21.7%, *p* = 0.086). When analyzed without stratification by indication, splenectomy showed no impact on OS (median 42.7 vs. 42.2 months, *p* = 0.665) or DFS (median 32.1 vs. 35.4 months, *p* = 0.138). However, stratification by indication revealed divergent prognostic effects: splenectomy for peritoneal implants independently predicted worse DFS (OR = 17.814, 95% CI: 3.025–104.894, *p* = 0.001), while splenectomy for hilar invasion was protective (OR = 0.136, 95% CI: 0.025–0.736, *p* = 0.021). PCI independently predicted both OS (OR = 1.150 per point, *p* = 0.006) and DFS (OR = 1.166 per point, *p* < 0.001). Primary tumor type was not independently prognostic after adjusting for PCI (OS *p* = 0.345, DFS *p* = 0.163). **Conclusions**: Splenectomy during CRS + HIPEC was associated with prolonged intensive care and hospital stays without increasing major complications. Peritoneal implant-related splenectomy predicts worse DFS, likely reflecting extensive disease burden, while hilar invasion-related splenectomy is protective, possibly reflecting more complete regional clearance achieved during en bloc resection to attain CC-0. Given the retrospective nature of this study and the heterogeneous patient population, these findings should be interpreted with caution. Further prospective research with larger, more homogeneous patient cohorts is warranted to definitively establish the long-term implications of splenectomy in CRS + HIPEC procedures.

## 1. Introduction

Cytoreductive surgery (CRS) combined with hyperthermic intraperitoneal chemotherapy (HIPEC) is one of the surgical methods used in patients with peritoneal carcinomatosis (PC). Its benefits have been demonstrated particularly in appendiceal PC cases [1] while its efficacy in other origins remains a topic of debate [2]. Moreover, although no randomized trial has been performed for diffuse malignant peritoneal mesothelioma, the beneficial effect of CRS + HIPEC is widely accepted. Its applications have further expanded to include various malignancies with peritoneal spread, including colorectal, gastric and pseudomyxoma peritonei. However, given the life-threatening complications of this procedure, patients’ peculiarities should be considered in the selection of this procedure [3,4,5,6,7]. In recent studies, the morbidity rates in patients undergoing CRS + HIPEC ranged from approximately 12% to 60%, while mortality rates ranged from approximately 0.9% to 5.8% [8].

Splenectomy may be necessary in peritoneal carcinomatosis (PC) due to hematologic, direct, or peritoneal dissemination, as well as following iatrogenic injuries. A recent study involving patients with epithelial ovarian cancer undergoing CRS with concurrent splenectomy due to splenic invasion found no difference in postoperative prognosis compared to other patient populations [9]. Another study evaluating patients with advanced-stage epithelial ovarian cancer reported that splenectomy was associated with more aggressive surgical interventions, higher rates of reoperation, blood transfusions, postoperative infections, and prolonged ICU stays, although disease-free survival (DFS) and overall survival (OS) rates were comparable between groups [10].

Among patients undergoing CRS, splenectomy was associated with increased minor complications, leading to recommendations for spleen preservation when feasible [11]. Similarly, data from PC patients revealed higher rates of infectious complications, pancreatic fistula, and intestinal perforation in those undergoing splenectomy, identifying it as a poor perioperative risk indicator [12]. Moreover, it has been shown that splenectomy performed during CRS + HIPEC may induce hematotoxic effects, and that granulocyte counts may increase, particularly following the use of oxaliplatin [13,14]. Another analysis highlighted that splenectomy was more frequent in patients with higher peritoneal cancer index (PCI) scores and elevated BMI undergoing CRS + HIPEC [15].

The impact of splenectomy on survival remains more controversial. Several studies have reported that cancer patients exhibit altered immune responses following splenectomy [16]. The most prominent theories supporting this observation primarily emphasize the spleen’s central role in the immune system, and secondarily, the potential delay of adjuvant therapy in already vulnerable patients due to major morbidity associated with splenectomy [17].

Two main mechanisms may explain the potential impact of splenectomy on survival. The spleen exerts a dual role in tumor immunology, influencing both immune activation and suppression depending on the host status [16,18]. While some studies suggest reduced immunosuppression and enhanced cytotoxic activity after splenectomy, others report increased regulatory T cell activity and metastasis [19,20,21,22,23]. Clinically and experimentally, inferior survival outcomes or immunosuppression have been observed in gastric and pancreatic cancers, suggesting that immune dysregulation after splenectomy may contribute to poorer prognosis, particularly in conditions like peritoneal carcinomatosis [24]. As another example in patients with ovarian cancer undergoing CRS + HIPEC, splenectomy performed due to invasion or hematologic dissemination did not influence long-term survival outcomes, which were instead dependent on chemotherapy sensitivity [25]. The specific impact of splenectomy during CRS + HIPEC procedures in malignant peritoneal mesothelioma and gastric cancers has not yet been specifically evaluated in the literature.

While prior studies have explored the implications of splenectomy in CRS + HIPEC, its effect on postoperative morbidity in PC patients remains unclear [26]. This study aims to evaluate the impact of splenectomy on postoperative morbidity in patients with PC undergoing CRS + HIPEC.

## 2. Materials and Methods

### 2.1. Design

The study was initiated following the approval of the ethics committee with the reference number 2023/06/679 dated 4 April 2023 at Kosuyolu Training and Research Hospital. Records of 149 patients who underwent CRS + HIPEC at the Department of Gastroenterological Surgery, Kosuyolu Training and Research Hospital, between 1 January 2018, and 31 December 2021, were examined. Patients with histologically confirmed colorectal cancer, pseudomyxoma peritoneii, ovarian cancer, gastric cancer or peritoneal malignant mesothelioma cancer diagnosis were included in the study and retrospectively screened. Informed consent was not obtained due to the retrospective nature of the study.

Patients who underwent palliative or emergency surgery, had incomplete perioperative or early postoperative clinical/laboratory data, or did not attend follow-up visits after discharge were excluded from the study to ensure data completeness. Additionally, patients with Eastern Cooperative Oncology Group scores ≥ 3 were excluded because of contraindications. Regarding disease origins, patients with PCI > 18 for colon cancer, patients with PCI > 10 for gastric cancer, and patients with sarcomatoid malign mesothelioma were also excluded. Lastly, patients who were previously diagnosed with extra-abdominal metastasis, centralization, and mesenteric root involvement were excluded to avoid incomplete cytoreductions. The CRS + HIPEC was performed according to the open technique described by Sugarbaker et al. [27].

### 2.2. Surgical Procedures and Indications for Splenectomy

Splenectomy was performed when one or more of the following conditions were present: (1) direct tumor invasion of the splenic parenchyma or hilum, (2) extensive disease on the splenic capsule that could not be cleared with local resection, (3) involvement of splenic flexure requiring en bloc resection, (4) iatrogenic injury during mobilization of adjacent structures, or (5) to facilitate exposure for comprehensive cytoreduction of the left upper quadrant peritoneum or diaphragm. The decision to perform splenectomy was made intraoperatively based on these findings.

For survival analysis, these indications were categorized into three groups: hilar tumor invasion (indication #1), peritoneal implants (indications #2, #3, #5), and iatrogenic injury (indication #4). Cases with overlapping features were categorized based on the primary indication requiring the most extensive resection.

Peritonectomy was performed only in patients with evident peritoneal metastases, and was not applied prophylactically. The procedures were carried out according to the standard peritonectomy techniques defined by Sugarbaker, including pelvic, diaphragmatic, and paracolic peritoneal stripping when indicated [27].

Following complete cytoreduction, HIPEC was administered using the open coliseum technique with continuous perfusion for 60 min. Disease-specific chemotherapy regimens were categorized into oxaliplatin-based and cisplatin-based protocols according to established guidelines. For colorectal cancer and pseudomyxoma peritonei: Intraperitoneal oxaliplatin 360–460 mg/m^2^ combined with intravenous 5-fluorouracil 400 mg/m^2^ and leucovorin 20 mg/m^2^ at 42 °C for 60 min. For gastric cancer, ovarian cancer, and malignant mesothelioma: Intraperitoneal cisplatin 50–100 mg/m^2^ combined with doxorubicin 15–50 mg/m^2^ at 41–43 °C for 60 min. Drug dosing was individualized based on patient body surface area and comorbidities. Inflow and outflow temperatures were continuously monitored and maintained within target range. Perfusion was performed using a roller pump with flow rates adjusted to maintain adequate circulation throughout the peritoneal cavity [28].

### 2.3. Study Endpoints

The primary endpoints of this study were the evaluation of the impact of splenectomy on OS, defined as the time from CRS + HIPEC procedure to death from any cause or last follow-up, and DFS, defined as the time from CRS + HIPEC procedure to first documented recurrence, progression, or death from any cause. Secondary endpoints included the comparison of postoperative morbidity and complications between splenectomy and non-splenectomy groups using the Clavien–Dindo classification system, with major complications defined as Clavien–Dindo Grade ≥ 3.

### 2.4. Measurements

The following were measured and recorded: operation duration, intravenous (IV) fluid and transfusion given during the operation, types of fluids administered, hypothermia or hyperthermia, amount of bleeding, use of catecholamines during the operation, body temperatures during the perioperative period, fluid intake and output, medications and dosages used in HIPEC, PCI scores during surgery, and complete cytoreduction scores. Inadequate cytoreduction was defined based on tumor type-specific thresholds: CC-3 (residual disease > 2.5 cm) for ovarian cancer, and CC-2 or higher (residual disease > 2.5 mm) for colorectal, gastric, pseudomyxoma peritonei, and mesothelioma. Patients with inadequate cytoreduction were excluded, as HIPEC was not administered in these cases [29]. In addition to patients’ surgical histories, records of resections and indications for splenectomy during their current surgeries were documented.

Patients’ surgical site infections were defined according to the Centers for Disease Control 1988 classification of surgical site infections modified in 2017 [30,31] and confirmed with culture results in all patients. Mobilization was implemented in all possible patients starting from the first postoperative day. Respiratory physiotherapy was initiated as early as possible after extubation. Urinary catheters were removed during admission to the ward if hemodynamic stabilization was achieved after urine output, and there were no pathologies requiring hourly monitoring. Central venous catheters were removed as early as possible after oral intake was established if peripheral IV cannulation was present. Drains placed routinely in parasplenic, pelvic, Morrison’s pouch, and subdiaphragmatic areas were removed as early as possible after clinical evaluation. Patients who were not vaccinated preoperatively and underwent splenectomy received meningococcal, pneumococcal, and influenza vaccines on postoperative day 15 unless contraindicated.

During the postoperative follow-up of patients, records of early or late metabolic, neurological, respiratory, surgical, and all other complications were extracted. Records of treatments administered for these complications, whether patients underwent repeat surgical procedures, and whether they developed sequelae after recovery were noted. Surviving patients were contacted by phone for survival analysis. Records were kept regarding whether patients received chemotherapy during postoperative follow-up and the number of cycles received, as well as whether they experienced recurrence during follow-up intervals for routine check-ups.

### 2.5. Statistical Analysis

Statistical analysis of the data was performed using the IBM SPSS Statistics for Windows, version 23.0 (IBM Corp., Armonk, NY, USA). Categorical measurements were summarized as counts and percentages. The normality of the variables was assessed using the Shapiro–Wilk test. Normally distributed continuous variables were expressed as mean ± standard deviation, while non-normally distributed variables were presented as median (interquartile range). The chi-squared test was utilized for comparisons of categorical variables. In cases where groups did not adhere to a normal distribution, the Mann–Whitney U test was employed. Survival analyses were conducted using Kaplan–Meier analysis and Log Rank tests. Multivariate Cox proportional hazards regression models were used to identify independent predictors of overall survival and disease-free survival, adjusting for potential confounding variables including age, gender, BMI, primary tumor type, PCI, and splenectomy indication. A statistical significance level of 0.05 was considered for all tests.

## 3. Results

Among the 149 patients included in the study, 78 (52.3%) had colorectal cancer, 24 (16.1%) had pseudomyxoma peritonei, 20 (13.4%) had gastric cancer, 18 (12.1%) had ovarian cancer, and 9 (6.0%) had malignant peritoneal mesothelioma. Splenectomy was performed in 52 (34.9%) patients, while 97 (65.1%) patients did not undergo splenectomy.

When comparing demographic and clinical parameters, gender distribution showed no significant difference between splenectomy and non-splenectomy groups (*p* = 0.376).

Primary malignancy distribution differed significantly between groups (*p* < 0.001), with colorectal cancer predominant in the non-splenectomy group (64.0%) compared to the splenectomy group (30.8%). Conversely, pseudomyxoma peritonei (26.9% vs. 10.3%) and malignant mesothelioma (13.4% vs. 2.1%) were more frequent in the splenectomy group. Comorbidities did not affect splenectomy rates. The median Peritoneal Cancer Index was significantly higher in the splenectomy group (9 vs. 3, *p* = 0.010). HIPEC regimen differed between groups, with oxaliplatin more commonly used in the non-splenectomy group (74.9% vs. 57.6%) and cisplatin more frequent in the splenectomy group (42.4% vs. 25.1%, *p* = 0.038).

Splenectomy group patients underwent more extensive surgical procedures, including peritonectomy (86.6% vs. 38.2%, *p* < 0.001), diaphragmatic peritonectomy (80.8% vs. 24.7%, *p* < 0.001), pelvic peritonectomy (59.7% vs. 28.9%, *p* < 0.001), total colectomy (26.9% vs. 2.1%, *p* < 0.001), colon resection (59.7% vs. 50.5%, *p* = 0.009), gastrectomy (25.0% vs. 12.4%, *p* = 0.049), Glisson capsule resection (36.5% vs. 7.3%, *p* < 0.001), distal pancreatectomy (7.7% vs. 0%, *p* = 0.014), and small intestine resection (30.7% vs. 14.4%, *p* = 0.017). Ostomy formation was more common in the splenectomy group, with ileostomy being the predominant type (88.4% ileostomy vs. 11.6% colostomy in splenectomy group, *p* = 0.041).

Intraoperative complications were more frequent in the splenectomy group (9.6% vs. 1.1%, *p* = 0.048), primarily bladder injury. Splenectomy patients had longer operations (10 [5–16] vs. 6 [3–14] h, *p* < 0.001), higher mean albumin transfusion (0.32 ± 0.10 vs. 0.17 ± 0.04 vials, *p* < 0.002), and extended ICU (1.5 [1–21] vs. 1 [1–20] days, *p* < 0.001) and hospital stays (12 [5–43] vs. 9 [4–77] days, *p* = 0.005).

Postoperatively, splenectomy patients experienced significantly higher rates of hepatotoxicity (27.0% vs. 4.2%, *p* < 0.001), pleural effusion (25.0% vs. 4.1%, *p* < 0.001), pancreatic fistula (15.4% vs. 1.1%, *p* = 0.001), nephrotoxicity (21.2% vs. 7.2%, *p* = 0.012), and pneumothorax (7.7% vs. 0%, *p* = 0.013). Disease recurrence and progression for CCS1 patients was significantly more common in the splenectomy group (44.2% vs. 26.8%, *p* = 0.031) (Table 1).

During OS and DFS analysis, 3 patients with early postoperative mortality were excluded and remaining 146 patients were analyzed. OS analysis showed no gender differences between survivors and non-survivors (*p* = 0.510). Primary tumor distribution showed no significant difference (*p* = 0.063), though gastric cancer was more prevalent among non-survivors (26.1% vs. 10.6%). Comorbidities showed no impact on survival. OS was lower in patients who underwent ostomy formation. (*p* = 0.014), with more colostomies among non-survivors (26.1% vs. 4.9%). Non-survivors had significantly longer operations (8.25 [6.00–11.50] vs. 6.00 [5.00–9.00] h, *p* = 0.030) and hospital stays (13.00 [9.00–22.00] vs. 10.00 [7.00–13.00] days, *p* = 0.015).

Specific complications were significantly more common in non-survivors, including encephalopathy (8.7% vs. 0.0%, *p* = 0.001), paraplegia (4.3% vs. 0.0%, *p* = 0.020), and cellulitis (8.7% vs. 0.8%, *p* = 0.014) (Table 2).

During DFS analysis, 146 patients were evaluated (101 with recurrence, 45 without recurrence). Primary tumor distribution and demographics showed no differences between groups. Smoking history was unexpectedly more common in the non-recurrence group (17.8% vs. 5.9%, *p* = 0.025).

Patients without recurrence underwent significantly more extensive surgical procedures, including peritonectomy (68.9% vs. 48.5%, *p* = 0.022), total colectomy (24.4% vs. 5.0%, *p* < 0.001), total gastrectomy (22.2% vs. 9.9%, *p* = 0.046), and splenectomy (46.7% vs. 29.7%, *p* = 0.047). Correspondingly, these patients had longer operative times (8.25 [6.00–12.00] vs. 6.00 [5.00–8.00] h, *p* < 0.001) and hospital stays (12.00 [9.00–15.00] vs. 9.00 [7.00–13.00] days, *p* = 0.002), reflecting more aggressive cytoreductive efforts.

Patients with recurrence had higher PCI scores (5 vs. 3, *p* = 0.002) and greater transfusion requirements, including albumin (0.40 ± 0.08 vs. 0.12 ± 0.04 vials, *p* < 0.001), packed red blood cells (1.31 ± 0.22 vs. 0.61 ± 0.11 units, *p* = 0.008), and fresh frozen plasma (2.04 ± 0.30 vs. 0.67 ± 0.12 units, *p* = 0.002). CCS 0 rates were higher in those without recurrence (86.7% vs. 96.0%, *p* = 0.038) (Table 3).

Cox regression analyses for OS showed that demographic factors including age, gender, BMI and complications associated with mortality were not significant predictors. In univariate analysis, splenectomy indication was significant (overall *p* = 0.117), with peritoneal implant splenectomy associated with increased mortality risk (OR = 3.679, 95% CI: 1.224–11.060, *p* = 0.020), while hilar invasion and iatrogenic splenectomy showed no significant associations. Postoperative complications (nephrotoxicity, hepatotoxicity, pneumothorax, pleural effusion, pancreatic fistula) showed no significant associations. Hospitalization duration was significant in univariate analysis (OR = 1.055, 95% CI: 1.014–1.098, *p* = 0.008).

Multivariate Cox regression analysis for OS included primary tumor type, complete cytoreduction score, PCI, splenectomy indication, and hospital length of stay. Primary tumor type showed no significant association with OS (overall *p* = 0.345), with all individual diagnostic categories remaining non-significant in multivariate modeling. Pseudomyxoma peritonei could not be evaluated due to zero mortality events in this group.

PCI emerged as a significant independent predictor of OS when modeled continuously (OR = 1.150 per 1-point increase, 95% CI: 1.041–1.270, *p* = 0.006), indicating that each unit increase in PCI was associated with a 15% increase in mortality risk. Hospital length of stay remained an independent predictor (OR = 1.058 per day, 95% CI: 1.005–1.112, *p* = 0.030), with each additional day increasing mortality risk by approximately 5.8%.

Splenectomy indication showed no overall significant association with OS in multivariate analysis (*p* = 0.121). However, when examined by specific indication, peritoneal implant splenectomy demonstrated a borderline association with increased mortality risk (OR = 4.505, 95% CI: 0.939–21.620, *p* = 0.060), though this did not reach statistical significance. Hilar invasion splenectomy showed no association with OS (OR = 0.448, 95% CI: 0.078–2.582, *p* = 0.369), and iatrogenic splenectomy could not be estimated due to zero events. Complete cytoreduction score showed no significant association with OS in multivariate analysis (Table 4).

Cox regression analyses for DFS showed no significant associations with age, gender, BMI, complications or primary tumor type. Also HIPEC-related and splenectomy-associated complications showed no association. Splenectomy indication was significant in both univariate and multivariate analyses (*p* < 0.001). In univariate analysis, splenectomy for peritoneal implants was associated with significantly increased recurrence risk (OR = 25.146, 95% CI: 5.410–116.886, *p* < 0.001), while iatrogenic splenectomy and hilar invasion showed no significant associations. PCI analyzed as a continuous variable was an independent predictor of recurrence in univariate (OR = 1.080, *p* = 0.003) analysis. Complete cytoreduction score showed borderline significance in univariate analysis (*p* = 0.050).

Multivariate Cox regression analysis for DFS included primary tumor type, splenectomy indication, complete cytoreduction score, and PCI (continuous). Primary tumor type showed no overall significant association with DFS (*p* = 0.163), though gastric cancer demonstrated a borderline trend toward increased recurrence risk (OR = 3.081, 95% CI: 0.845–11.230, *p* = 0.088).

Splenectomy indication emerged as a highly significant independent predictor of DFS (overall *p* < 0.001). Peritoneal implant-related splenectomy was strongly associated with increased recurrence risk (OR = 17.814, 95% CI: 3.025–104.894, *p* = 0.001), indicating an approximately 18-fold increase in recurrence compared to patients without splenectomy. In contrast, hilar dissection-related splenectomy was independently associated with improved DFS (OR = 0.136, 95% CI: 0.025–0.736, *p* = 0.021), representing an 86% reduction in recurrence risk.

PCI analyzed as a continuous variable was a significant independent predictor of recurrence (OR = 1.166 per 1-point increase, 95% CI: 1.066–1.276, *p* = 0.001), with each unit increase in PCI associated with a 16.6% increase in recurrence risk. Complete cytoreduction score showed no significant association with DFS in multivariate analysis (Table 5).

**Figure 1:**
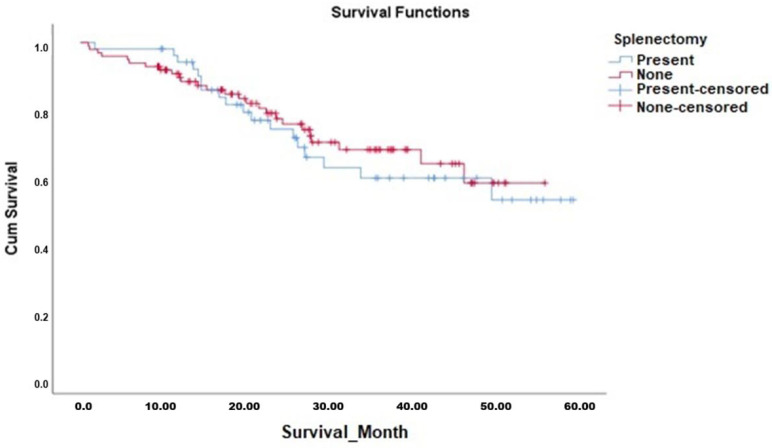
Overall Survival Analysis of All Cases Depending on Splenectomy.

**Figure 2:**
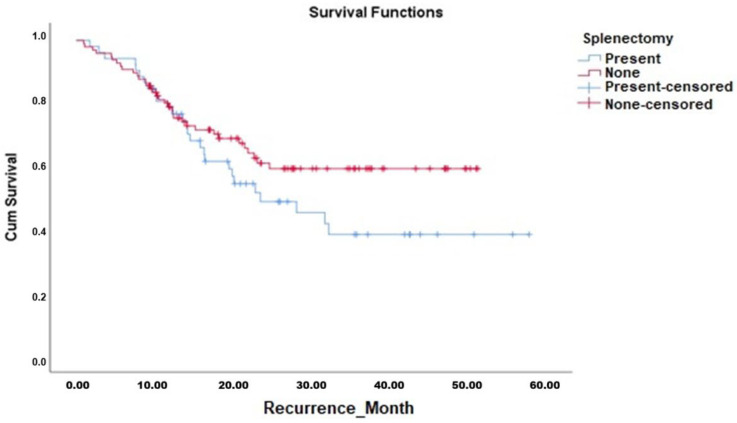
Disease-Free Survival Analysis of All Cases Depending on Splenectomy.

Kaplan–Meier survival analyses demonstrated that the OS duration in patients who underwent splenectomy (median 42.7 months, 95% CI 36.7–48.7 months) was comparable to those who did not undergo splenectomy (median 42.2 months, 95% CI 37.9–46.5 months). The Log-Rank test confirmed no significant difference between the survival curves (χ^2^ = 0.187, *p* = 0.665). Similarly, DFS analysis showed no significant difference between patients who underwent splenectomy (median 32.1 months, 95% CI 25.8–38.5 months) and those who did not (median 35.4 months, 95% CI 31.2–39.6 months; Log-Rank χ^2^ = 2.202, *p* = 0.138). The Kaplan–Meier curves for OS and DFS are presented in Figure 1 and Figure 2, respectively, with corresponding survival data detailed in Table 6.

## 4. Discussion

This study aimed to analyze the morbidity and survival outcomes following splenectomy in patients undergoing CRS + HIPEC. Our findings demonstrated that splenectomy was significantly associated with prolonged ICU and hospital stay durations. While complications such as nephrotoxicity, hepatotoxicity, pneumothorax, pleural effusion, and pancreatic fistula were more frequently observed in patients who underwent splenectomy, the rates of major complications remained statistically similar between the splenectomy and non-splenectomy groups. Correspondingly, OS rates showed no significant differences between the two groups. However, when stratified by surgical indication, splenectomy demonstrated divergent prognostic impacts on DFS. In patients who underwent splenectomy due to the presence of peritoneal implants, DFS was independently and strongly associated with recurrence (OR = 17.814, *p* = 0.001), representing an approximately 18-fold increased risk. Conversely, splenectomy for hilar tumor invasion was independently associated with improved DFS (OR = 0.136, *p* = 0.021), likely reflecting the more extensive perisplenic lymphadenectomy required in these cases. This association was not observed in cases where the indication was iatrogenic injury, which showed a protective trend without reaching statistical significance. Additionally, peritoneal disease burden quantified as continuous PCI emerged as an independent predictor of both OS (OR = 1.150, *p* = 0.006) and DFS (OR = 1.166, *p* = 0.001), while primary tumor type showed no independent prognostic significance after adjusting for PCI and surgical factors (OS *p* = 0.345, DFS *p* = 0.163).

Non-hematological cancer cases have been associated with splenectomy as an additional surgical procedure, which has been shown to be associated with additional surgical complications and a poor prognostic effect. Studies evaluating the relationship between splenectomy and complications in patients with PC reveal that splenectomy in CRS + HIPEC procedures is associated with an increased frequency of pulmonary complications and a higher incidence of Grade 3–4 complications. In our study, although individual pulmonary complications were observed more frequently in the splenectomy group (pneumothorax, pleural effusion), multivariate analyses showed that major complications (Clavien–Dindo Grade ≥ 3) were not independently associated with splenectomy. Splenectomy was independently associated with prolonged hospitalization (OR = 1.058 per day, *p* = 0.030), likely reflecting overall surgical complexity and disease burden rather than representing a direct causal effect.

Saxena et al. conducted a comprehensive study including 936 patients to investigate the outcomes of splenectomy in all patients undergoing CRS + HIPEC. It was observed that patients who underwent splenectomy, approximately 418 patients, had higher PCIs, and underwent more aggressive surgical procedures such as major peritonectomy, partial gastrectomy, or colectomy. It was shown that patients who underwent splenectomy had a higher incidence of pancreatic fistula, infectious complications, intra-abdominal collection development, bleeding, intestinal fistula, and sepsis. An increase in all grade 3 and 4 complications, as well as prolonged hospital and ICU stays, were observed. Similarly, in the present study, similar to the report by Saxena et al., the need for splenectomy increased when the PCI was ≥6. Additionally, longer hospital and ICU stays in splenectomy patients were also demonstrated in our study. However, the isolated or overall increase in complications mentioned in the study by Saxena et al. was not observed to be associated with splenectomy in our study [12].

In a study by Angeles et al. involving 992 patients who underwent HIPEC, splenectomy requirement was found to be associated with the development of gastric perforation, high BMI, and high PCI in 533 patients who underwent CRS. However, in our study group, no patients with gastric perforation were observed. Similarly to the study by Angeles et al., our study also showed a significant association between higher peritoneal disease burden and increased need for splenectomy [15].

Regarding studies on patient survival, only studies associated with CRS + HIPEC in ovarian cancers have been identified in the literature. In a study involving 28 patients with ovarian cancer who underwent CRS + HIPEC, it was shown that there was no difference between patients who underwent splenectomy and those who did not [9]. However, this study included only patients with splenectomized ovarian cancers and was primarily designed to evaluate hematological complications. Therefore, it would not be appropriate to make comparisons between the study and our study. In a study by Said et al., splenectomy was performed in 99 patients with advanced ovarian cancer, and their data were compared with those of patients who did not undergo splenectomy. Although patients who underwent splenectomy required more aggressive surgical interventions, surgical procedure reoperations, and blood transfusions and had more postoperative infections and longer ICU stays, similar rates of DFS and OS were found in these patients [10]. In our study, no increased need for splenectomy, specifically in patients with ovarian cancers, was observed. When all patient groups were examined, it was shown that patients who underwent splenectomy had higher PCI scores, required more transfusions, and had longer ICU and hospital stays; this is similar to the findings in the study by Said et al. [10]. However, in our study, the rates of postoperative surgical site infections and reoperation were similarly distributed between the groups. Disease-specific subgroup analysis for ovarian cancer was not feasible due to limited sample size (*n* = 18 with only 2 deaths), precluding reliable survival comparisons stratified by splenectomy status. Nevertheless, in the overall cohort analysis, splenectomy indication emerged as a critical prognostic determinant: splenectomy performed for peritoneal implants was independently associated with worse DFS (OR = 17.814, *p* = 0.001), while splenectomy for hilar invasion requiring extensive lymphadenectomy was protective (OR = 0.136, *p* = 0.021).

In our study, no relationship was found between early postoperative mortality (<90 days) and splenectomy. When analyzed without stratification by indication, splenectomy showed no independent association with OS or DFS. However, stratification by surgical indication revealed critical prognostic differences: splenectomy for peritoneal implants independently predicted markedly increased recurrence (OR = 17.814, *p* = 0.001), while splenectomy for hilar tumor invasion was protective (OR = 0.136, *p* = 0.021). The association between peritoneal implant splenectomy and recurrence primarily reflects the extent of peritoneal disease burden rather than the splenectomy procedure itself. Patients requiring splenectomy for peritoneal implants had significantly higher PCI scores (median 9 vs. 3, *p* = 0.010) and more extensive peritoneal involvement, which independently drives recurrence risk.

Several limitations should be considered when interpreting our findings. The retrospective design limits our ability to control for all confounding factors. Patient selection for CRS + HIPEC is individualized based on tumor characteristics and clinical condition. Our cohort included patients with different primary malignancies, each with different biological behavior and prognosis. This heterogeneity makes it difficult to separate the effects of splenectomy from the effects of underlying disease.

The rarity of some conditions prevented reliable disease-specific analyses (Supplementary Materials). Therefore, survival outcomes may reflect tumor biology and disease burden rather than splenectomy itself. For this reason, we analyzed the entire cohort to maintain statistical power while acknowledging this limitation. In view of these limitations, we analyzed the widest possible range of parameters to achieve the most comprehensive evaluation of patient survival and postoperative complications. Further evaluations with more homogenous and disease specific studies are needed.

While our stratified analysis by splenectomy indication revealed divergent prognostic effects (implant indicated splenectomy predicting worse DFS vs. hilar invasion-related splenectomy being protective), we cannot definitively establish causality. The finding that hilar invasion splenectomy improves DFS while peritoneal implant splenectomy worsens outcomes cannot fully separate whether benefits derive from completeness of lymphadenectomy, en bloc resection technique, or unmeasured confounders such as intrinsic tumor biology. Prospective studies with standardized lymph node dissection protocols, pathologic assessment of lymph node yields and metastatic involvement, and ideally propensity score matching or randomized comparison would be required to definitively establish whether lymphadenectomy completeness causally determines outcomes.

Even when analyzing the overall cohort without disease-specific stratification, sample sizes within splenectomy indication subgroups were limited, resulting in wide confidence intervals and limited statistical power for detecting small effect sizes. The protective trend observed with iatrogenic splenectomy (OR = 0.133, *p* = 0.120), while reassuring, did not reach statistical significance and should be interpreted cautiously given the small sample size.

Also exploring complications for CRS + HIPEC in a heterogenous cohorts should be carefully evaluated. As for disease specific regimens used in protocols, tendency for complications may cause different results. Our cohort consists of two different hipec protocols. OS and DFS analysis stratified by tumor type was not feasible due to small sample sizes in most diagnostic categories (gastric *n* = 20, ovarian *n* = 18, mesothelioma *n* = 9) and insufficient outcome events. Notably, pseudomyxoma peritonei demonstrated zero mortality events (excellent prognosis), precluding any statistical comparison. This limitation also depends on both temporal factors (patients operated in 2021–2022 with <2 years follow-up) and the favorable oncologic outcomes achieved in certain diagnostic subgroups.

Also these limitations reflect the natural challenges of studying rare peritoneal surface malignancies, where even multi-year single-center cohorts struggle to achieve adequate sample sizes for disease-specific analyses. Multicenter collaborative studies or international registry data would be required to adequately power subgroup analyses by tumor type.

This study has several notable strengths. The stratified analysis by splenectomy indication represents a novel approach not previously reported in peritoneal surface malignancy literature, revealing prognostic differences obscured in aggregate analyses. Complete perioperative data collection, standardized HIPEC protocols, and detailed documentation of splenectomy indications strengthen the validity of our findings. The analysis of PCI as a continuous variable rather than dichotomized cutoffs provides superior prognostic stratification and validates the Sugarbaker index as a quantitative biomarker. Finally, inclusion of both morbidity and survival outcomes provides comprehensive evaluation of splenectomy’s impact on perioperative and oncologic endpoints.

## 5. Conclusions

This study evaluated the impact of splenectomy in patients undergoing CRS + HIPEC on morbidity and mortality. Our findings demonstrate that splenectomy significantly increases ICU and hospital stay durations in patients. However, splenectomy was not found to be a risk factor for the development of major complications in CRS + HIPEC cases. Additionally, when analyzed without stratification by surgical indication, splenectomy did not show any significant effect on overall survival or disease-free survival.

However, stratification by splenectomy indication revealed critical prognostic differences previously obscured in aggregate analyses. While PCI was the main predictor of both OS and DFS as expected; DFS rates in patients who underwent splenectomy for peritoneal implants were found to be markedly lower, while splenectomy for hilar tumor invasion requiring extensive perisplenic lymphadenectomy was strongly protective.

The protective effect observed with hilar invasion splenectomy represents a hypothesis-generating finding requiring prospective validation. Specifically, randomized controlled trials or propensity score-matched studies are needed to distinguish whether improved disease-free survival derives from complete regional clearance to achieve CC-0 in cases of hilar involvement, or disease-specific systematic lymphadenectomy beyond what is necessary for macroscopic cytoreduction. Such studies should incorporate standardized splenectomy indication definitions, protocolized extent of perisplenic dissection, comprehensive pathologic examination with lymph node yields and stations documented, and ideally randomized comparison of standard versus extended lymphadenectomy in patients with hilar involvement.

Furthermore, investigating the increased occurrence of pulmonary complications after splenectomy and assessing factors associated with delayed recovery in these patients through quality-of-life scoring could help explain the lack of differences in survival or recurrence timing despite the increase in hospital and ICU stays. Conducting such studies could also lead to a clearer understanding of the situation.

## Figures and Tables

**Table 1:** Clinicopathologic Variables Compared Based on Splenectomy Presence.

		Splenectomy		
		None (*n* = 97)	Present (*n* = 52)	*p* †
		*n* (%)	*n* (%)	
Gender	Female	54 (55.7)	25 (48)	0.376
	Male	43 (44.3)	27 (52)	
Diagnosis	Colorectal Cancer	62 (64)	16 (30.8)	<0.001 ***
	Gastric Cancer	12 (12.3)	8 (15.4)	
	Ovary Cancer	11 (11.3)	7 (13.4)	
	Pseudomyxoma Peritonei	10 (10.3)	14 (26.9)	
	Malignant Mesothelioma	2 (2.06)	7 (13.4)	
Diabetes	None	84 (86.6)	43 (82.7)	0.092
	Present	13 (13.4)	9 (17.3)	
CAD	None	91 (93.8)	52 (100.0)	0.068
	Present	6 (6.2)	0 (0.00)	
Smoking	None	88 (90.7)	47 (90.4)	0.359
	Present	9 (9.3)	5 (9.6)	
COPD or Asthma	None	95 (97.9)	49 (94.2)	0.343
	Present	2 (2.1)	3 (5.8)	
Hypothyroidism	None	93 (95.9)	49 (94.2)	0.695
	Present	4 (4.1)	3 (5.8)	
Hypertension	None	70 (72.1)	41 (78.8)	0.372
	Present	27 (27.9)	11 (21.2)	
Radiotherapy History	None	91 (93.8)	52 (100.0)	0.092
	Present	6 (6.2)	0 (0.00)	
Chemotherapy History	None	32 (32.9)	16 (30.7)	0.782
	Present	65 (67.1)	36 (69.3)	
HIPEC Regimen	Oxaliplatin Based	72 (74.9)	30 (57.6)	0.038 *
	Cisplatin Based	25 (25.1)	22 (42.4)	
Peroperative Complication	None	96 (98.9)	47 (90.4)	0.048 *
	Ureter Injury	1 (1.1)	1 (1.9)	
	Diaphragma Injury	0 (0.0)	3 (5.8)	
	Bladder Injury	0 (0.0)	1 (1.9)	
Peroperative Colonic or Small Intestine Resection	None	95 (97.9)	52 (100)	0.297
	Present	2 (2.1)	0 (0)	
Peritonectomy	None	60 (61.8)	7 (13.4)	<0.001 ***
	Present	37 (38.2)	45 (86.6)	
Diaphragmatic Peritonectomy	None	73 (75.3)	10 (19.2)	<0.001 ***
	Present	24 (24.7)	42 (80.8)	
Pelvic Peritonectomy	None	69 (71.1)	21 (40.3)	<0.001 ***
	Present	28 (28.9)	31 (59.7)	
TAHBSO ^1^	None	18 (33.3)	12 (48.0)	0.212
	Present	36 (66.7)	13 (52.0)	
Gastrectomy	None	85 (87.6)	39 (75.0)	0.049 *
	Present	12 (12.4)	13 (25.0)	
Colon Resection	None	48 (49.5)	21 (40.3)	0.009 **
	Present	49 (50.5)	31 (59.7)	
Total Colectomy	None	95 (97.9)	38 (73.1)	<0.001 ***
	Present	2 (2.1)	14 (26.9)	
Right HC	None	81 (83.5)	43 (82.6)	0.899
	Present	16 (16.5)	9 (17.4)	
Left HC	None	94 (96.9)	46 (88.4)	0.065
	Present	3 (3.1)	6 (11.6)	
Subtotal Colectomy	None	96 (98.9)	52 (100)	1
	Present	1 (1.1)	0 (0.00)	
Glisson Capsule Resection	None	90 (92.7)	33 (63.5)	<0.001 ***
	Present	7 (7.3)	19 (36.5)	
Liver Metastasectomy	None	92 (94.8)	48 (92.3)	0.719
	Present	5 (5.2)	4 (7.7)	
Distal Pancreatectomy	None	97 (100)	48 (92.3)	0.014 **
	Present	0 (0)	4 (7.7)	
Ostomy Formation	Ileostomy	14 (63.6)	23 (88.4)	0.041 *
	Colostomy	8 (36.4)	3 (11.6)	
Rectosigmoid Resection	None	65 (67.1)	32 (61.5)	0.504
	Present	32 (32.9)	20 (38.5)	
Small Intestine Resection	None	83 (85.5)	36 (69.3)	0.017 *
	Present	14 (14.4)	16 (30.7)	
Postoperative Mortality	None	95 (97.9)	51 (98.1)	1
	Mortality within 30 days	2 (2.1)	1 (1.9)	
Complication	Minor	76 (78.3)	34 (65.3)	0.086
	Major	21 (21.7)	18 (34.7)	
Reoperation	None	90 (92.7)	45 (86.5)	0.245
	Present	7 (7.3)	7 (13.5)	
Recurrence	None	71 (73.2)	29 (55.8)	0.031 *
	Present	26 (26.8)	23 (44.2)	
Resection	None	2 (2.1)	0 (0.00)	0.543
	Present	95 (97.9)	52 (100.0)	
Nephrotoxicity	None	90 (92.8)	41 (78.8)	0.012
	Present	7 (7.2)	11 (21.2)	
Hepatotoxicity	None	93 (95.8)	38 (73)	<0.001 ***
	Present	4 (4.2)	14 (27)	
Neutropenia	None	97 (100)	51 (98)	0.171
	Present	0 (0)	1 (2)	
Pneumothorax	None	97 (100)	48 (92.3)	0.013 *
	Present	0 (0)	4 (7.7)	
Pleural Effusion	None	93 (95.9)	39 (75)	<0.001 ***
	Present	4 (4.1)	13 (25)	
Anastomosis Leakage ^2^	None	76 (96.2)	34 (94.5)	0.653
	Present	3 (3.8)	2 (5.5)	
Chylous Fistula	None	88 (90.7)	50 (96.1)	0.33
	Present	9 (9.3)	2 (3.9)	
Intestinal Fistula	None	94 (96.9)	51 (98)	1
	Present	3 (3.1)	1 (2)	
Pancreatic Fistula	None	96 (98.9)	44 (84.6)	0.001 ***
	Present	1 (1.1)	8 (15.4)	
Biliary Tract Fistula	None	96 (98.9)	52 (100)	1
	Present	1 (1.1)	0 (0)	
Ostomy Necrosis ^3^	None	23 (100)	25 (96.1)	0.342
	Present	0 (0)	1 (3.9)	
Postoperative Ileus	None	89 (91.7)	51 (98)	0.162
	Present	8 (8.3)	1 (2)	
Cellulitis	None	96 (98.9)	50 (96.1)	0.278
	Present	1 (1.1)	2 (3.9)	
Encephalopathy	None	97 (100)	50 (96.1)	0.12
	Present	0 (0)	2 (3.9)	
Paraplegia	None	96 (98.9)	52 (100)	1
	Present	1 (1.1)	0 (0)	
Pneumonia	None	91 (93.8)	44 (84.6)	0.081
	Present	6 (6.2)	8 (15.4)	
Bleeding	None	91 (93.8)	48 (92.3)	0.39
	Medical Treatment	4 (4.1)	1 (1.9)	
	Surgical Treatment	2 (2.1)	3 (5.8)	
Arrhythmia	None	94 (96.9)	50 (96.1)	1
	Present	3 (3.1)	2 (3.9)	
Rectovaginal Fistula ^1^	None	53 (98.1)	25 (100)	0.493
	Present	1 (1.9)	0 (0)	
SSI	None	73 (75.3)	37 (71.2)	0.587
	Present	24 (24.7)	15 (28.8)	
		**Median (IQR)**	**Median (IQR)**	***p* ‡**
Age	Years	56 (53–59)	57 (53–60)	0.578
BMI	kg/m^2^	26.64 (25.91–28.48)	27.48 (25.38–29.69)	0.873
Operation Duration	Hours	6 (3–14)	10 (5–16)	<0.001 ***
ICU duration	Days	1 (1–20)	1.5 (1–21)	<0.001 ***
Hospitalization Duration	Days	9 (4–77)	12 (5–43)	0.005 **
PCI		3 (3–4)	9 (2–14)	0.010 **
		**Mean ± sd**	**Mean ± sd**	***p* Γ**
Albumin Transfusion	Per Vial	0.17 ± 0.04	0.32 ± 0.10	<0.002 **
PRBC Transfusion	Unit	0.80 ± 0.11	0.92 ± 0.23	0.326
FFP Transfusion	Unit	1.14 ± 0.16	0.97 ± 0.26	0.563

^1^ Only female patients included. ^2^ Patients who underwent gastrointestinal anastomosis during surgery were included. ^3^ Patients who underwent ileostomy or colostomy during surgery were included. CAD: Coronary artery disease; COPD: Chronic obstructive pulmonary disease; HIPEC: hyperthermic intraperitoneal chemotherapy, TAHBSO: Total Abdominal Hysterectomy and Bilateral Salpingo-Oophorectomy; HC: Hemicolectomy; PCI: Peritoneal Cancer Index; BMI: Body Mass Index, PRBC: Packed Red Blood Cells; FFP: Fresh Frozen Plasma; SSI: Surgical Site Infection; ICU: Intensive Care Unit, * *p* < 0.05, ** *p* < 0.01, *** *p* < 0.001, † Chi-squared test, ‡ Mann–Whitney U test, Γ: Independent Samples T Test.

**Table 2:** Clinicopathologic Variables Compared Based on Overall Survival.

		Overall Survival		
		Alive (*n* = 123)	Not Alive (*n* = 23)	*p* †
		*n* (%)	*n* (%)	
Gender	Female	68 (55.3)	11 (47.8)	0.51
	Male	55 (44.7)	12 (52.2)	
Diagnosis	Colorectal Cancer	62 (50.4)	14 (60.9)	0.063
	Gastric Cancer	13 (10.6)	6 (26.1)	
	Ovary Cancer	16 (13.0)	2 (8.7)	
	Pseudomyxoma Peritonei	24 (19.5)	0 (0.0)	
	Malignant Mesothelioma	8 (6.5)	1 (4.3)	
Diabetes	None	104 (84.6)	21 (91.3)	0.397
	Present	19 (15.4)	2 (8.7)	
CAD	None	118 (95.9)	22 (95.7)	0.95
	Present	5 (4.1)	1 (4.3)	
Smoking	None	112 (91.1)	20 (87.0)	0.54
	Present	11 (8.9)	3 (13.0)	
COPD or Asthma	None	120 (97.6)	21 (91.3)	0.13
	Present	3 (2.4)	2 (8.7)	
Hypothyroidism	None	117 (95.1)	23 (100.0)	0.279
	Present	6 (4.9)	0 (0.0)	
Hypertension	None	93 (75.6)	17 (73.9)	0.862
	Present	30 (24.4)	6 (26.1)	
Radiotherapy History	None	118 (95.9)	21 (91.3)	0.917
	Present	5 (4.1)	2 (8.7)	
Chemotherapy History	None	42 (34.1)	5 (21.7)	0.242
	Present	81 (65.9)	18 (78.3)	
HIPEC Regimen	Oxaliplatin Regimen	94 (76.4)	17 (73.9)	0.796
	Cisplatin Regimen	29 (23.5)	6 (26.1)	
Peroperative Complication	None	119 (96.7)	22 (95.7)	0.107
	Ureter Injury	2 (1.6)	0 (0.0)	
	Diaphragma Injury	2 (1.6)	0 (0.0)	
	Bladder Injury	0 (0.0)	1 (4.3)	
Peroperative Colonic or Small Intestine Resection	None	2 (1.6)	0 (0.0)	0.538
	Present	121 (98.4)	23 (100.0)	
Peritonectomy	None	57 (46.3)	9 (39.1)	0.524
	Present	66 (53.7)	14 (60.9)	
TAHBSO ^1^	None	22 (48.9)	1 (12.5)	0.056
	Present	23 (51.1)	7 (87.5)	
Gastrectomy	None	106 (86.2)	17 (73.9)	0.311
	Subtotal Gastrectomy	2 (1.6)	1 (4.3)	
	Total Gastrectomy	15 (12.2)	5 (21.7)	
Colon Resection	None	83 (67.5)	13 (56.5)	0.309
	Present	40 (32.5)	10 (43.5)	
Total Colectomy	None	110 (89.4)	20 (87.0)	0.727
	Present	13 (10.6)	3 (13.0)	
Right HC	None	103 (83.7)	18 (78.2)	0.522
	Present	20 (16.3)	5 (21.8)	
Left HC	None	116 (95.1)	20 (90.9)	0.432
	Present	6 (4.9)	2 (9.1)	
Subtotal Colectomy	None	122 (99.2)	23 (100.0)	0.664
	Present	1 (0.8)	0 (0.0)	
Liver Metastasectomy	None	116 (94.3)	21 (91.3)	0.582
	Present	7 (5.7)	2 (8.7)	
Glisson Capsule Resection	None	100 (81.3)	20 (87.0)	0.515
	Present	23 (18.7)	3 (13.0)	
Distal Pancreatectomy	None	119 (96.7)	23 (100.0)	0.381
	Present	4 (3.3)	0 (0.0)	
Ostomy Formation	None	87 (70.7)	12 (52.2)	0.014 *
	Ileostomy	30 (24.4)	5 (21.7)	
	Colostomy	6 (4.9)	6 (26.1)	
Rectosigmoid Resection	None	84 (68.3)	12 (52.2)	0.135
	Present	39 (31.7)	11 (47.8)	
Small Intestine Resection	None	100 (81.3)	17 (73.9)	0.415
	Present	23 (18.7)	6 (26.1)	
PCI	<6	80 (65.0)	12 (52.2)	0.241
	≥6	43 (35.0)	11 (47.8)	
Complication	Minor	93 (75.6)	16 (69.6)	0.541
	Major	30 (24.4)	7 (30.4)	
CCS	0	115 (93.4)	21 (91.3)	0.703
	1	8 (6.6)	2 (8.7)	
Reoperation	None	113 (91.9)	19 (82.6)	0.166
	Present	10 (8.1)	4 (17.4)	
Nephrotoxicity	None	110 (89.4)	18 (78.3)	0.135
	Present	13 (10.6)	5 (21.7)	
Hepatotoxicity	None	108 (87.8)	20 (87.0)	0.91
	Present	15 (12.2)	3 (13.0)	
Neutropenia	None	122 (99.2)	23 (100.0)	0.664
	Present	1 (0.8)	0 (0.0)	
Pneumothorax	None	120 (97.6)	22 (95.7)	0.607
	Present	3 (2.4)	1 (4.3)	
Pleural Effusion	None	111 (90.2)	18 (78.3)	0.1
	Present	12 (9.8)	5 (21.7)	
Anastomosis Leakage ^2^	None	93 (97.9)	16 (88.9)	0.058
	Present	2 (2.1)	2 (11.1)	
Chylous Fistula	None	113 (91.9)	22 (95.7)	0.528
	Present	10 (8.1)	1 (4.3)	
Intestinal Fistula	None	121 (98.4)	21 (91.3)	0.057
	Present	2 (1.6)	2 (8.7)	
Pancreatic Fistula	None	117 (95.1)	20 (87.0)	0.135
	Present	6 (4.9)	3 (13.0)	
Biliary Tract Fistula	None	122 (99.2)	23 (100.0)	0.664
	Present	1 (0.8)	0 (0.0)	
Ostomy Necrosis ^3^	None	36 (100.0)	10 (90.9)	0.067
	Present	0 (0.0)	1 (9.1)	
Postoperative Ileus	None	117 (95.1)	21 (91.3)	0.46
	Present	6 (4.9)	2 (8.7)	
Cellulitis	None	122 (99.2)	21 (91.3)	0.014 *
	Present	1 (0.8)	2 (8.7)	
Encephalopathy	None	123 (100.0)	21 (91.3)	0.001 *
	Present	0 (0.0)	2 (8.7)	
Paraplegia	None	123 (100.0)	22 (95.7)	0.020 *
	Present	0 (0.0)	1 (4.3)	
Pneumonia	None	115 (93.5)	19 (82.6)	0.081
	Present	8 (6.5)	4 (17.4)	
Bleeding	None	115 (93.5)	21 (91.3)	0.206
	Medical Treatment	3 (2.4)	2 (8.7)	
	Surgical Treatment	5 (4.1)	0 (0.0)	
Arrhythmia	None	120 (97.6)	22 (95.7)	0.607
	Present	3 (2.4)	1 (4.3)	
Rectovaginal Fistula ^1^	None	65 (98.4)	11 (100.0)	0.681
	Present	1 (1.6)	0 (0.0) 0 (0.0)	
SSI	None	88 (71.5)	13 (56.5)	0.152
	Present	35 (28.5)	10 (43.5)	
		**Median (IQR)**	**Median (IQR)**	***p* ‡**
Age	Years	56.00 (48.00–62.00)	57.00 (44.00–64.00)	0.593
BMI	kg/m^2^	26.64 (24.22–30.47)	28.11 (25.39–31.51)	0.769
Operation Duration	Hours	6.00 (5.00–9.00)	8.25 (6.00–11.50)	0.030 *
Hospitalization Duration	Days	10.00 (7.00–13.00)	13.00 (9.00–22.00)	0.015 *
PCI	Score	3 (2–5)	5 (3–14)	0.019 *
		**Mean ± sd**	**Mean ± sd**	***p* Γ**
Albumin Transfusion	Per Vial	0.07 ± 0.03	0.45 ± 0.09	0.045 *
PRBC Transfusion	Unit	0.71 ± 0.13	1.82 ± 0.28	0.462
FFP Transfusion	Unit	1.03 ± 0.15	1.43 ± 0.33	0.111

^1^ Only female patients included. ^2^ Patients who underwent gastrointestinal anastomosis during surgery were included. ^3^ Patients who underwent ileostomy or colostomy during surgery were included. CAD: Coronary artery disease; COPD: Chronic obstructive pulmonary disease; HIPEC: hyperthermic intraperitoneal chemotherapy, TAHBSO: Total Abdominal Hysterectomy and Bilateral Salpingo-Oophorectomy; HC: Hemicolectomy; PCI: Peritoneal Cancer Index; CCS: Complete Cytoreduction Score, BMI: Body Mass Index, PRBC: Packed Red Blood Cells; FFP: Fresh Frozen Plasma; SSI: Surgical Site Infection; * *p* < 0.05, † Chi-squared test, ‡ Mann–Whitney U test, Γ Independent Samples *t* Test.

**Table 3:** Clinicopathologic Variables Compared Based on Recurrence.

		Disease-Free Survival		
		Recurrence (*n* = 101)	Non-Recurrence (*n* = 45)	*p* †
		*n* (%)	*n* (%)	
Gender	Female	58 (57.4)	21 (46.7)	0.228
	Male	43 (42.6)	24 (53.3)	
Diagnosis	Colorectal Cancer	54 (53.5)	22 (48.9)	0.787
	Gastric Cancer	11 (10.9)	8 (17.8)	
	Ovary Cancer	12 (10.9)	6 (13.3)	
	Pseudomyxoma Peritonei	18 (17.8)	6 (13.3)	
	Malignant Mesothelioma	6 (5.9)	3 (6.7)	
Diabetes	None	86 (85.1)	39 (86.7)	0.809
	Present	15 (14.9)	6 (13.3)	
CAD	None	97 (96.0)	43 (95.6)	0.892
	Present	4 (4.0)	2 (4.4)	
Smoking	None	95 (94.1)	37 (82.2)	0.025 *
	Present	6 (5.9)	8 (17.8)	
COPD or Asthma	None	98 (97.0)	43 (95.6)	0.651
	Present	3 (3.0)	2 (4.4)	
Hypothyroidism	None	97 (96.0)	43 (95.6)	0.892
	Present	4 (4.0)	2 (4.4)	
Hypertension	None	73 (72.3)	37 (82.2)	0.198
	Present	28 (27.7)	8 (17.8)	
Radiotherapy History	None	95 (95.0)	44 (97.8)	0.437
	Present	5 (5.0)	1 (2.2)	
Chemotherapy History	None	36 (35.6)	11 (24.4)	0.181
	Present	65 (64.4)	34 (75.6)	
Peroperative Complication	None	99 (98.0)	42 (93.3)	0.391
	Ureter Injury	1 (1.0)	1 (2.2)	
	Diaphragma Injury	1 (1.0)	1 (2.2)	
	Bladder Injury	0 (0.0)	1 (2.2)	
Peroperative Colonic or Small Intestine Resection	None	2 (2.0)	0 (0.0)	0.342
	Present	99 (98.0)	45 (100.0)	
Peritonectomy	None	52 (51.5)	31 (68.9)	0.022 *
	Present	49 (48.5)	14 (31.1)	
TAHBSO ^1^	None	17 (47.2)	6 (35.3)	0.413
	Present	19 (52.8)	11 (64.7)	
Gastrectomy	None	90 (89.1)	33 (73.3)	0.046*
	Subtotal Gastrectomy	1 (1.0)	2 (4.4)	
	Total Gastrectomy	10 (9.9)	10 (22.2)	
Colon Resection	None	71 (70.3)	25 (55.6)	0.083
	Present	30 (29.7)	20 (44.4)	
Splenectomy	None	71 (70.3)	24 (53.3)	0.047 *
	Present	30 (29.7)	21 (46.7)	
Total Colectomy	None	96 (95.0)	34 (75.6)	<0.001 ***
	Present	5 (5.0)	11 (24.4)	
Right HC	None	81 (80.2)	40 (88.9)	0.198
	Present	20 (19.8)	5 (11.1)	
Left HC	None	97 (96.0)	41 (91.1)	0.227
	Present	4 (4.0)	4 (8.9)	
Subtotal Colectomy	None	100 (99.0)	45 (100.0)	0.503
	Present	1 (1.0)	0 (0.0)	
Glisson Capsule Resection	None	86 (85.1)	34 (75.6)	0.162
	Present	15 (14.9)	11 (24.4)	
Liver Metastasectomy	None	94 (93.1)	43 (95.6)	0.564
	Present	7 (6.9)	2 (4.4)	
Distal Pancreatectomy	None	97 (96.0)	45 (100.0)	0.176
	Present	4 (4.0)	0 (0.0)	
Ostomy Formation	None	75 (74.3)	24 (53.3)	0.042 *
	Ileostomy	21 (20.8)	14 (31.1)	
	Colostomy	5 (5.0)	6 (13.3)	
Rectosigmoid Resection	None	71 (70.3)	25 (55.6)	0.083
	Present	30 (29.7)	20 (44.4)	
Small Intestine Resection	None	81 (80.2)	36 (80.0)	0.978
	Present	20 (19.8)	9 (20.0)	
HIPEC Regimen	Oxaliplatin Based	79 (77.4)	32 (71.1)	0.353
	Cisplatin Based	23 (22.6)	13 (28.9)	
CCS	0	97	39	0.038 *
	1	4	6	
Complication	Minor	27 (26.7) 74 (73.3)	10 (22.2) 35 (77.8)	0.563
	Major	74 (73.3) 27 (26.7)	35 (77.8) 10 (22.2)	
Reoperation	None	90 (89.1)	42 (93.3)	0.423
	Present	11 (10.9)	3 (6.7)	
Nephrotoxicity	None	91 (90.1)	37 (82.2)	0.181
	Present	10 (9.9)	8 (17.8)	
Hepatotoxicity	None	92 (91.1)	36 (80.0)	0.06
	Present	9 (8.9)	9 (20.0)	
Neutropenia	None	100 (99.0)	45 (100.0)	0.503
	Present	1 (1.0)	0 (0.0)	
Pneumothorax	None	98 (97.0)	44 (97.8)	0.798
	Present	3 (3.0)	1 (2.2)	
Pleural Effusion	None	91 (90.1)	38 (84.4)	0.325
	Present	10 (9.9)	7 (15.6)	
Anastomosis Leakage ^2^	None	77 (97.5)	32 (94.1)	0.377
	Present	2 (2.5)	2 (5.9)	
Chylous Fistula	None	95 (94.1)	40 (88.9)	0.274
	Present	6 (5.9)	5 (11.1)	
Intestinal Fistula	None	99 (98.0)	43 (95.6)	0.4
	Present	2 (2.0)	2 (4.4)	
Pancreatic Fistula	None	96 (95.0)	41 (91.1)	0.361
	Present	5 (5.0)	4 (8.9)	
Biliary Tract Fistula	None	100 (99.0)	45 (100.0)	0.503
	Present	1 (1.0)	0 (0.0)	
Ostomy Necrosis ^3^	None	27 (100.0)	19 (95.0)	0.24
	Present	0 (0.0)	1 (5.0)	
Postoperative Ileus	None	95 (94.1)	43 (95.6)	0.714
	Present	6 (5.9)	2 (4.4)	
Cellulitis	None	100 (99.0)	43 (95.6)	0.174
	Present	1 (1.0)	2 (4.4)	
Encephalopathy	None	101 (100.0)	43 (95.6)	0.033 *
	Present	0 (0.0)	2 (4.4)	
Paraplegia	None	100 (99.0)	45 (100.0)	0.503
	Present	1 (1.0)	0 (0.0)	
Pneumonia	None	91 (90.1)	43 (95.6)	0.268
	Present	10 (9.9)	2 (4.4)	
Bleeding	None	93 (92.1)	43 (95.6)	0.291
	Medical Treatment	3 (3.0)	2 (4.4)	
	Surgical Treatment	5 (5.0)	0 (0.0)	
Arrhythmia	None	100 (99.0)	42 (93.3)	0.052
	Present	1 (1.0)	3 (6.7)	
Rectovaginal Fistula ^1^	None	55 (98.3)	21 (100.0)	0.538
	Present	1 (1.7)	0 (0.0)	
SSI	None	72 (71.3)	29 (64.4)	0.408
	Present	29 (28.7)	16 (35.6)	
		**Median (IQR)**	**Median (IQR)**	***p* ‡**
Age	Years	56 (48–63)	55 (44–59)	0.363
BMI	kg/m^2^	26.64 (23.88–30.74)	27.48 (24.76–30.42)	0.715
Operation Duration	Hours	6.00 (5.00–8.00)	8.25 (6.00–12.00)	<0.001 ***
Hospitalization Duration	Days	9.00 (7.00–13.00)	12.00 (9.00–15.00)	0.002 **
PCI	Score	5 (3–12)	3 (2–5)	0.002 **
		**Mean ± sd**	**Mean ± sd**	***p* Γ**
Albumin Transfusion	Per Vial	0.40 ± 0.08	0.12 ± 0.04	<0.001 ***
PRBC Transfusion	Unit	1.31 ± 0.22	0.61 ± 0.11	0.008 **
FFP Transfusion	Unit	2.04 ± 0.30	0.67 ± 0.12	0.002 **

^1^ Only female patients included. ^2^ Patients who underwent gastrointestinal anastomosis during surgery were included. ^3^ Patients who underwent ileostomy or colostomy during surgery were included. CAD: Coronary artery disease; COPD: Chronic obstructive pulmonary disease; HIPEC: hyperthermic intraperitoneal chemotherapy, TAHBSO: Total Abdominal Hysterectomy and Bilateral Salpingo-Oophorectomy; HC: Hemicolectomy; PCI: Peritoneal Cancer Index; CCS: Complete Cytoreduction Score, BMI: Body Mass Index, PRBC: Packed Red Blood Cells; FFP: Fresh Frozen Plasma; SSI: Surgical Site Infection; * *p* < 0.05, ** *p* < 0.01, *** *p* < 0.001, † Chi-squared test, ‡ Mann–Whitney U test, Γ: Independent Samples *t* Test.

**Table 4:** Cox regression analyses of parameters associated with overall survival.

Variables		Univariate		Multivariate	
		*p*	OR [95% CI]	*p*	OR [95% CI]
Age		0.719	1.007 (0.968–1.048)	-	-
Gender		0.511	1.349 (0.553–3.291)	-	-
BMI		0.426	1.038 (0.947–1.138)	-	-
Diagnosis	Colorectal Cancer	0.57	-	0.345	-
	Gastric Cancer	0.214	2.044 (0.662–6.314)	0.120	3.253 (0.737–14.368)
	Ovary Cancer	0.463	0.554 (0.114–2.688)	0.398	0.419 (0.056–3.144)
	PSP	0.998	NE *	0.997	NE *
	MM	0.561	0.554 (0.064–5.792)	0.498	0.437 (0.040–4.788)
Splenectomy	None	0.117	-	0.121	-
	Iatrogenic	0.999	0.000	0.999	NE *
	Peritoneal Implant	0.020	3.679 [1.224–11.060]	0.060	4.505 (0.939–21.620)
	Hilar Invasion	0.879	0.901 [0.235–3.454]	0.369	0.448 (0.078–2.582)
Nephrotoxicity		0.144	2.350 (0.748–7.390)	-	-
Hepatotoxicity		0.910	1.080 (0.286–4.076)	-	-
Pneumothorax		0.612	1.818 (0.181–18.287)	-	-
Pleural Effusion		0.110	2.569 (0.809–8.164)	-	-
Pancreatic Fistula		0.151	2.925 (0.676–12.656)	-	-
HIPEC Drug Regimen		0.796	1.144 (0.413–3.171)	-	-
Hospitalization, days		0.008	1.055 (1.014–1.098)	0.030 **	1.058 (1.005–1.112)
CCS		0.704	1.369 (0.272–6.903)	0.166	0.167 (0.013–2.103)
PCI, Score		0.093	1.052 (0.992–1.116)	0.006 **	1.150 (1.041–1.270)

* NE: Not estimable due to zero or very few events in this category. OR: Odds ratio, 95% CI = 95% Confidence Interval for the Odds Ratio, BMI: Body Mass Index, CCS: Complete Cytoreduction Score PCI: Peritoneal Carcinomatosis Index, PSP: Psudomyxoma Peritoneii, MM: Malignant Mesothelioma. ** *p* < 0.01

**Table 5:** Cox regression analyses of parameters associated with disease-free survival.

Variables		Univariate		Multivariate	
		*p*	OR [95% CI]	*p*	OR [95% CI]
Age		0.485	0.989 (0.960–1.020)	-	-
Gender		0.23	1.542 (0.761–3.123)	-	-
BMI		0.762	1.012 (0.939–1.089)	-	-
Diagnosis	Colorectal Cancer	0.792	-	0.163	-
	Gastric Cancer	0.273	1.785 (0.633–5.035)	0.088	3.081 (0.845–11.230)
	Ovary Cancer	0.715	1.227 (0.409–3.680)	0.625	0.688 (0.153–3.086)
	PSP	0.708	0.818 (0.287–2.335)	0.117	0.255 (0.046–1.409)
	MM	0.785	1.227 (0.282–5.348)	0.586	0.879 (0.081–4.147)
Splenectomy	None	<0.001	-	<0.001	-
	Iatrogenic	0.431	0.423 (0.049–3.613)	0.12	0.133 (0.011–1.690)
	Peritoneal Implant	<0.001	25.146 [5.410–116.886]	0.001	17.814 (3.025–104.894)
	Hilar Invasion	0.192	0.423 [0.116–1.543]	0.021	0.136 (0.025–0.736)
Nephrotoxicity		0.187	1.968 (0.720–5.376)	-	-
Hepatotoxicity		0.066	2.556 (0.939–6.954)	-	-
Pneumothorax		0.799	0.742 (0.075–7.338)	-	-
Pleural Effusion		0.329	1.676 (0.594–4.730)	-	-
Pancreatic Fistula		0.367	1.873 (0.479–7.333)	-	-
HIPEC Drug Regimen		0.354	1.459 (0.656–3.244)	-	-
Hospitalization		0.263	1.019 (0.986–1.052)	-	-
CCS		0.050	3.731 (0.998–13.946)	0.730	0.698 (0.090–5.402)
PCI, Score		0.003	1.080 (1.027–1.136)	<0.001	1.166 (1.066–1.276)

OR: Odds ratio, 95% CI = 95% Confidence Interval for the Odds Ratio, BMI: Body Mass Index, CCS: Complete Cytoreduction Score, PCI: Peritoneal Carcinomatosis Index, PSP: Psudomyxoma Peritoneii, MM: Malignant Mesothelioma.

**Table 6:** Data table regarding Disease-Free and Overall Survival Times.

	DFS Time (Months)	Standard Error	95% Confidence Interval
Splenectomy Present	32.135	3.256	(25.754–38.517)
No Splenectomy	35.409	2.147	(31.201–39.616)
General	36.498	2.032	(32.515–40.481)
	**OS Time (months)**	**Standard Error**	**%95 Confidence Interval**
Splenectomy Present	42.666	3.053	(36.682–48.650)
No Splenectomy	42.222	2.207	(37.897–46.547)
General	43.606	1.901	(39.880–47.333)

DFS: Disease-Free Survival, OS: Overall Survival.

## Data Availability

The data that support the findings of this study are available from TR S.B.U. Kosuyolu Yuksek Ihtisas Training and Research Hospital, but restrictions apply to the availability of these data, which were used under license for the current study and so are not publicly available. Data are, however, available from the authors upon reasonable request and with permission of TR S.B.U. Kosuyolu Yuksek Ihtisas Training and Research Hospital’s administration.

## Supplementary Materials

The following supporting information can be downloaded at https://www.mdpi.com/article/10.3390/jcm14228223/s1. Figure S1: OS analysis of Colorectal Cancer Patients; Figure S2: DFS analysis of Colorectal Cancer Patients; Figure S3: OS analysis of Gastric Cancer Patients; Figure S4: OS analysis of Ovary Cancer Patients; Figure S5: OS analysis of Malign Mesothelioma Patients.

## Abbreviations

CRS	Cytoreductive Surgery
HIPEC	Hyperthermic Intraperitoneal Chemotherapy
PC	Peritoneal Carcinomatosis
OS	Overall Survival
DFS	Disease-Free Survival
PCI	Peritoneal Cancer Index
ICU	Intensive Care Unit
