# Supplementary material for: The Effect of Splenectomy on Postoperative Morbidity and Survival in Patients with Peritoneal Carcinomatosis
Source: J Clin Med. 2025 Nov 20;14(22):8223. doi: 10.3390/jcm14228223 (PMC12653215; doi:10.3390/jcm14228223)
Supplement: Supplementary file 1 [file jcm-14-08223-s001.zip › jcm-3900360-supplementary.pdf]

Following reviewers' requests for tumor-type stratified survival analyses, we attempted disease-specific Kaplan–Meier curves for overall survival and disease-free survival. However, substantial limitations preclude reliable interpretation. Most diagnostic categories have critically small sample sizes (gastric n=20, ovarian n=18, mesothelioma n=9) that violate statistical guidelines requiring minimum 15-20 patients per group with ≥10 events. Several subgroups had zero or very few events, making statistical comparison impossible. Disease-free survival analysis was particularly constrained by insufficient recurrence events across most diagnostic subgroups.

These limitations result in wide confidence intervals, unstable survival estimates, and limited statistical power. The figures below are provided solely for transparency and should be interpreted with extreme caution. We strongly advise against drawing definitive conclusions from these underpowered analyses. For statistically robust inference, multivariate Cox regression with primary tumor type as a covariate (main manuscript Tables 4-5) provides a more appropriate approach by utilizing the full cohort while adjusting for diagnostic heterogeneity.

We would also like to share some information with you regarding one of the issues raised by Reviewer 1. Since our study specifically focused on splenectomy, we did not perform a regression analysis for major morbidity, as no significant association was observed. However, upon Reviewer 1's request, we evaluated the HIPEC regimens and found that oxaliplatin-based HIPEC was more frequently used in patients who underwent splenectomy. When we checked for potential bias related to HIPEC regimen selection, we found no significant association between the type of HIPEC regimen and the development of major complications ($p = 0.267$). Among the parameters showing statistical significance on major complication development were the completeness of cytoreduction score ($p = 0.042$), PCI score ($p = 0.030$), total colectomy ($p = 0.007$), intensive care unit duration ($p=0.005$), hospitalization ($p<0.001$), surgery duration ($p<0.001$) and bleeding volume ($p=0.016$). In addition to the HIPEC drug regimen, no significant results were found for HIPEC-related parameters such as pulse, body temperature, urine output, blood glucose, infused fluid volume, drained fluid volume, mean volume change, or transfusion records. We preferred to share these results here rather than include them in the manuscript, as adding these analyses or creating an additional table would considerably extend the length of the manuscript.

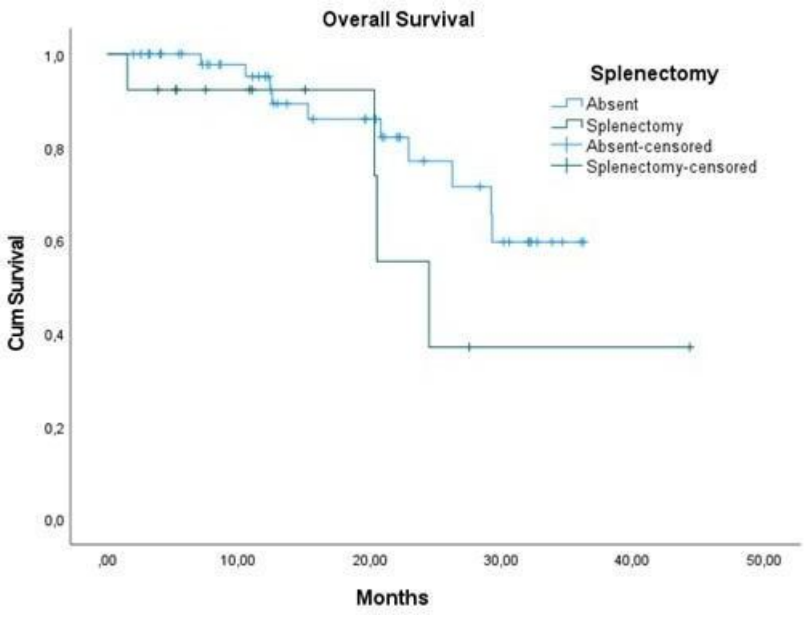

Supplementary Figure S1: OS analysis of Colorectal Cancer Patients.

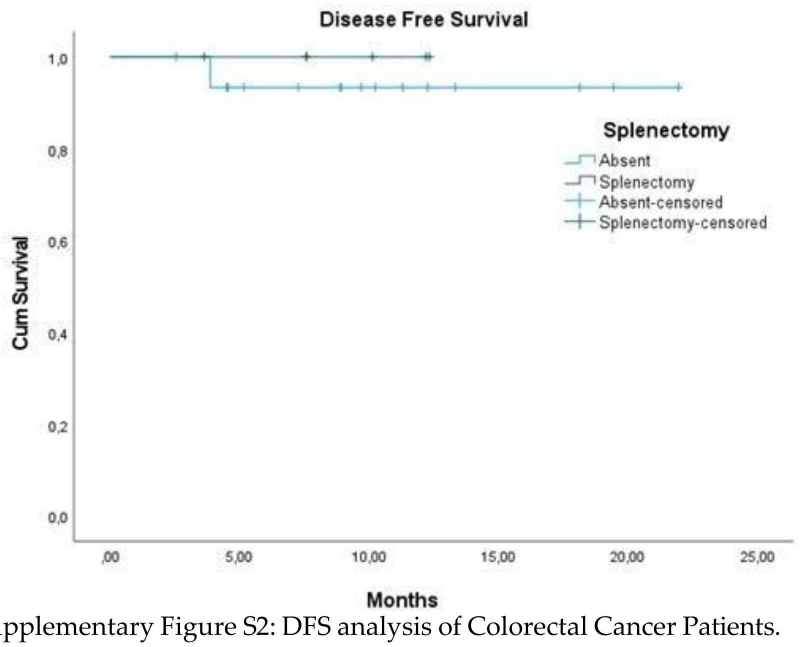

Supplementary Figure S2: DFS analysis of Colorectal Cancer Patients.

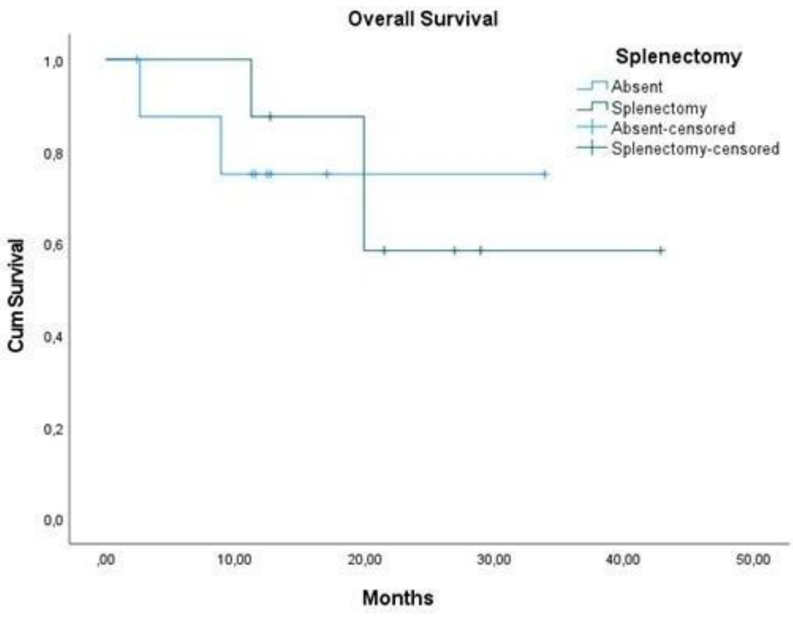

Supplementary Figure S3: OS analysis of Gastric Cancer Patients

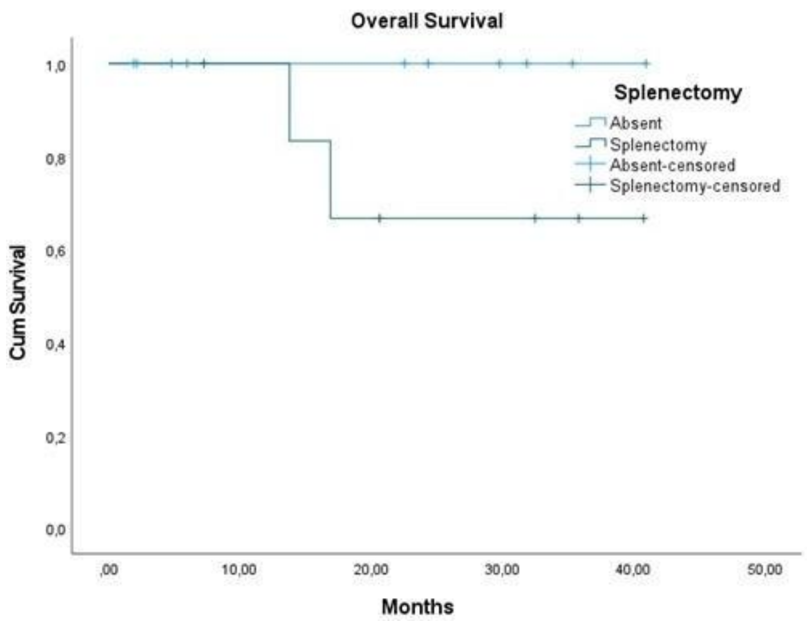

Supplementary Figure S4: OS analysis of Ovary Cancer Patients.

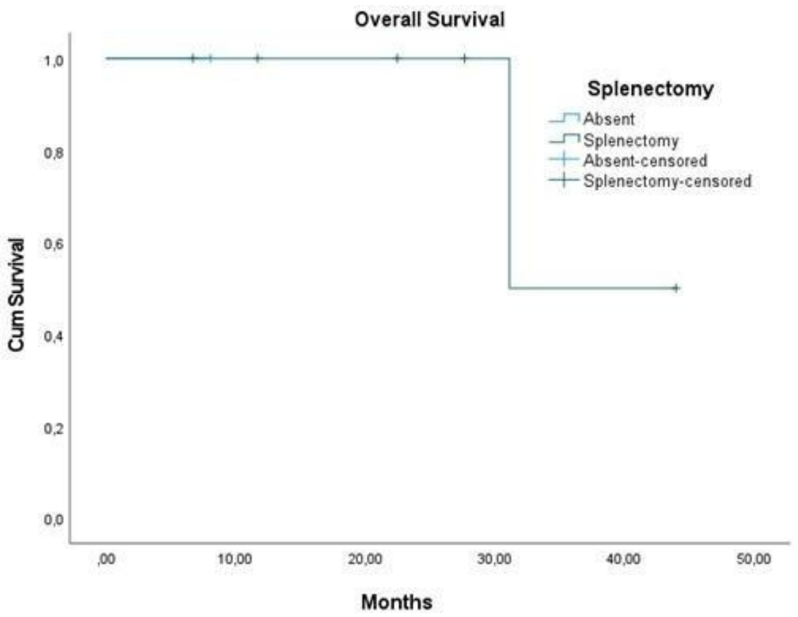

Supplementary Figure S5: OS analysis of Malign Mesothelioma Patients.
